# Analgesic Characteristics of Bupivacaine Alone and in Combination with Dexmedetomidine or Meperidine in Spinal Anesthesia during Cesarean Section: A Double-Blind Randomized Clinical Trial Study

**DOI:** 10.1155/2022/5111214

**Published:** 2022-07-18

**Authors:** Simin Azemati, Amir Zarghami, Reza Jouybar, Vida Naderi-boldaji

**Affiliations:** ^1^Anesthesiology and Critical Care Research Center, Shiraz University of Medical Sciences, Shiraz, Iran; ^2^Dena Hospital, Shiraz, Iran

## Abstract

**Background:**

Comparing bupivacaine's adjuvants in spinal anesthesia, we assessed the specific blocking characteristics and adverse effects of bupivacaine alone and in combination with dexmedetomidine or meperidine in spinal anesthesia during cesarean section.

**Methods:**

In this double-blind randomized clinical trial study, ninety pregnant women were divided into groups to receive 10 mg bupivacaine (group B), 10 mg bupivacaine with 5 *μ*g dexmedetomidine (group BD), or 10 mg bupivacaine with 10 mg meperidine (group BM) intrathecal. Patients were assessed for the quality of analgesia during operations. Durations of sensory and motor blocks and anesthesia-related complications were analyzed using SPSS 21, and *p* values <0.05 were considered statistically significant.

**Results:**

The onset of sensory and motor blocks was essentially the same in all treated groups. Block regression time was significantly prolonged in the BD group compared to the B and BM groups (*p* < 0.001). The duration of analgesia was significantly longer in the BD and BM groups than in the B group (*p* < 0.001). The level of sedation in the BD group was higher than in the B group. Shivering occurred in 40% of patients in the B group, which was significantly more than that of the BD (16.6%) and BM (33.3%) groups. Itching happened in 33.3% of women in the BM group which was statistically more than that of the B (3.33%) and BD (0) groups. The incidence of adverse effects was the same in all groups.

**Conclusion:**

The combination of bupivacaine with dexmedetomidine significantly prolonged sensory and motor regression time and duration of analgesia.

## 1. Introduction

Cesarean section is the most prevalent surgical procedure in the United States and accounts for more than 25% of all live births. The spinal anesthesia is widely used and is considered as an appropriate and safer method in the cesarean section than other techniques because it is simple to administer, induced by lower dose of drugs, and therefore, unlikely to produce systemic effects in the baby. It improves the neonatal outcome, decreases the risk of maternal pulmonary aspiration, and provides effective postoperative pain control. Some disadvantages of this technique are reduced duration of anesthesia and its association with a high incidence of hypotension during anesthesia [1, 2].

Bupivacaine is the most commonly-used local anesthetic in spinal anesthesia for cesarean section [3]. It is a long-acting local anesthetic and, compared to other local anesthetics, it has a limited transfer to the placenta. Administration of a single intrathecal low dose of bupivacaine for labor analgesia has been demonstrated and found to be effective [4]. Various adjuvants such as fentanyl [4], sufentanil [5], morphine [6], clonidine [7], and dexmedetomidine [8] have been added to intrathecal bupivacaine in local anesthesia to provide a prolonged duration of sensory block and reduce the dose of intrathecal local anesthetic, which can subsequently decrease the incidence of spinal-induced hypotension. Dexmedetomidine (DMT), centrally acting 2-selective agonist (*α*2-AR), has been reported to prolong the duration of spinal analgesia when adding to local anesthetic so it reduces the dose of intrathecal local anesthetics and the requirements for opioids in postoperative pain control [9–11]. It is proven that the combination of dexmedetomidine with bupivacaine 5% in lower abdominal surgery causes a longer sensory and motor block [12].

Therefore, we conducted this study to compare the specific blocking characteristics of bupivacaine in combination with dexmedetomidine or meperidine and to investigate whether these combinations would produce an appropriate sensory block for caesarian section and postoperative pain control or not.

## 2. Method and Materials

### 2.1. Study Design

This randomized double-blind clinical trial study was conducted on pregnant women presenting for elective cesarean section and requesting analgesia, and it was approved by the Ethics Committee (and registered in IRCT by a code of (IRCT2014100814372N4) of the Shiraz University of Medical Sciences, Shiraz, Iran.

### 2.2. Patients

Patients with ASA physical status I and II and uncomplicated term pregnancy of aa singleton fetus were included in the study. Exclusion criteria: patients with a positive history of cardiovascular or liver disease, renal failure, and seizure or other neurologic disorders, pregnancy-induced hypertension, contraindications to regional anesthesia, allergic reaction to the study agents, and patients who were unable to communicate or refused to participate.

### 2.3. Randomization and Blinding

To calculate the sample size, the comparison of means formula ([alpha] = 0.05 and [power] = 0.80) showed that at least 22 patients per study group were needed to detect an increase of 15 min difference between the mean duration of time for sensory regression to S1 segment (min) between the groups [13]. 90 patients were randomly assigned to three equal groups using block randomization in blocks of size 6 (list blocks were extracted from https://www.sealedenvelope.com): B group that received 2 ml bupivacaine 0.5% (10 mg), the BD group treated with 10 mg bupivacaine and 5 *μ*g dexmedetomidine, and the BM group that received 10 mg bupivacaine and 10 mg meperidine. The study solutions were prepared and coded by a nurse anesthetist who did not participate in the other parts of the study. Patients were prehydrated with 700–1000 mL of ringer lactate.

### 2.4. Surgical Procedures, Data Collection, and Outcomes

Spinal anesthesia was performed using a size 25G needle and intrathecal injection of the study agent through the L4/L5 intervertebral. Heart rate, blood pressure, and oxygen concentration were recorded every 5 minutes for the first 20 minutes after which they were taken at every 10 minutes interval by the end of surgery. The level of sensory block was assessed by a blinded anesthetist using the Pinprick test. The times of bilateral loss of sensation along the midclavicular line, duration of sensory block, time interval of intrathecal injection to a loss of sensation at T6 and the times from intrathecal injection to two dermatomes of sensory regression were recorded. The motor blockade, onset as well as regression, was evaluated concurrently with sensory blockade every 10 min after the spinal block, using a modified Bromage score 0–3 (0, no motor block; 1, unable to raise extended legs, able to move knees and feet; 2, unable to raise extended legs and move knees, able to move feet; and 3, complete motor block of the lower limbs) [14].

Pain intensity and duration were rated by the parturient using the visual pain score (VPS) ranging from 0 = pain free up to 10 = worst pain imaginable. VPS was recorded in the recovery room and thereafter, every time the patient expressed pain [14]. The end of analgesia was defined as the time when VPS was recorded at more than 4.

The Ramsay score was used for the assessment of sedation level in patients. It divides a patient's level of sedation into six categories ranging from severe agitation [1] to deep coma [5]. Systolic blood pressure and heart rate were recorded at the same intervals. Hypotension is defined as a fall in systolic blood pressure (SBP) of >30% of baseline value, and bradycardia as heart rate < 50 beats/min and these were treated with intravenous ephedrine (5 mg) and atropine (0.6 mg), respectively. Neonatal Apgar scores, as a means of rapid evaluation of the physical condition of infants, and umbilical venous blood pH were also recorded.

### 2.5. Statistical Analysis

Data was expressed as either the mean ± SEM, the median (interquartile rang), or numbers and percentages. Continuous variables in the demographic data of patients were analyzed using analysis of variance (ANOVA), repeated measure ANOVA, and the Kruskal–Wallis test. For categorical variables (ASA class, hypotension, bradycardia, and use of ephedrine, sedation scores, and Apgar score) the comparison was studied using the chi-squared test or the Fisher's exact test. Data were analyzed using SPSS 21 and *p* values <0.05 were considered statistically significant.

## 3. Results

### 3.1. Demographic and Baseline Clinical Characteristics of the Patients

**Figure 1:**
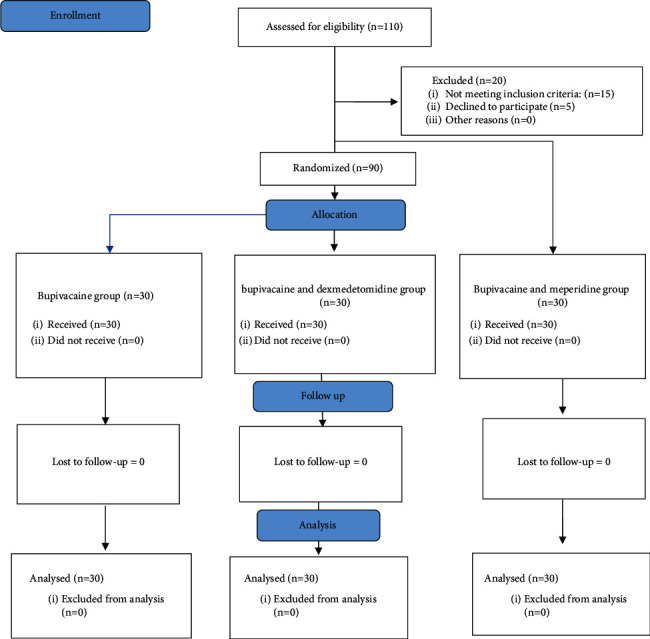
Consort follow diagram.

A total of ninety (90) patients in three equal groups participated throughout the study (Figure 1). Each group received 10 mg bupivacaine 0.5% intrathecal, then dexmedetomidine (10 mg) and meperidine (5 mg) were added to the bupivacaine in the BD and BM groups, respectively.

**Figure 2:**
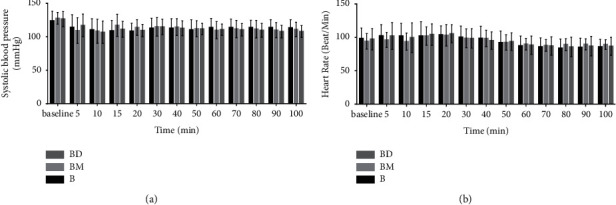
(a) Systolic blood pressure; (b) heart rate.

The hemodynamic parameters of the baseline heart rate, systolic blood pressure (SBP), and patient characteristics are shown in Table 1. There was no significant difference between study groups with respect to patient demographics such as age, weight, gestational age, ASA classification, and hemodynamic parameters. Systolic blood pressure (SBP) and heart rate (HR) were recorded separately for all 3 groups. Measurement times of vital results were taken every 5 minutes for 20 minutes and then every 10 minutes until surgery was completed. The first measurement time was the paranesthesia value; SBP was similar among the groups at all measurement times (Figure 2(a)). Similarly, HR values have also been found to be similar across all measurement times among groups (Figure 2(b)).

### 3.2. Spinal Block Characteristics and the Level of Sedation

Table 2 shows the block onset and regression times of the intrathecal agents and the level of sedation in different groups. The onset of sensory and motor block (min) was essentially the same in all groups. However, the time for sensory block to reach the T8 segment was different in the three groups. The BD group attained the T8 sensory block at a longer time compared to the B (*p* < 0.01) and BM (*p* < 0.05) groups. In comparison to the B and BM groups, the time for motor block regression to Bromage 0 was also significantly longer in the BD group (*p* < 0.01 and *p* < 0.05) respectively. The duration of analgesia was significantly longer in the BD and BM groups than in the B group (*p* < 0.01). The level of sedation in the BD group was higher than in the B and BM groups.

### 3.3. Safety and Adverse Effects

Table 3 shows the adverse events observed and recorded during the study. Hypotension, a fall in systolic blood pressure (SBP) of >30% of baseline value, and bradycardia, heart rate < 50 beats/min were noted as two of adverse events. The occurrence of hypotension was similar in all three groups in the way that two (6.67%) women in group B, 1 (3.33%) woman in group BD, and 2 (6.67%) women in group BD, had mild hypotension that was corrected with fluid administration. Bradycardia was not observed in the study groups.

The incidence of shivering in the B group was 40%, while 16.6% of patients in the BD group and 3.33% of the BM group experienced shivering. It means that the combination of dexmedetomidine or meperidine with bupivacaine could decrease the incidence of shivering. In relation to itching, this adverse event occurred in 10 (33.3%) of women in group BM, which was statistically more than that of the B (3.33%) and BD (0) groups.

### 3.4. Neonatal Outcomes

Apgar scores (at 1 and 5 min) and umbilical artery PH in the three groups were within normal values. The five minute Apgar score (9.80 ± 4.1) was significantly higher in the BD group than in the B (9.53 ± 0.51) and the BM (9.37 ± 0.49) groups, but other neonatal parameters showed no significant differences between the three groups (Table 4).

## 4. Discussion

To achieve the ideal regional block with a long duration of analgesia and to provide high-quality analgesia without side effects, several adjuvants (e.g. opioids [15], local anesthetics, and *α*2-adrenergic agonists, particularly clonidine) are added to local anesthetics. However, administration of opioids is associated with itching, drowsiness, nausea and vomiting, respiratory depression, or urinary retention [16].

It is proven that DEX as an effective adjuvant for regional anesthetic agents, increases the duration of spinal anesthesia and prolongs the duration of sensory [17] and motor block [18] and increases the quality of analgesia without neurologic sequelae when administered as an adjuvant to local anesthetics [19]. However, there is no proper consensus regarding the dose of drug to be used for proper blocks. Different doses varying from 3 to 15 mcg have been used as adjuvants to bupivacaine for spinal anesthesia [13].

This study was designed to compare the specific blocking characteristics, hemodynamic status, postoperative analgesia, and adverse effects of bupivacaine alone and in combination with dexmedetomidine or meperidine, and to investigate whether these combinations would produce an appropriate sensory block for caesarian section and postoperative pain control or not. Our findings revealed that the times for sensory and motor block regression were significantly longer in the bupivacaine-dexmedetomidine group as compared with both the bupivacaine–meperidine and bupivacaine groups. The supplementation of bupivacaine with a low-dose dexmedetomidine produces a significantly longer sensory and motor block than bupivacaine alone. Suppression of neuronal firing in the locus coeruleus through the hyperpolarization of noradrenergic neurons [17] in addition to inhibition of norepinephrine release and activity in the descending medullospinal noradrenergic pathway [20] are some probable mechanisms that facilitate analgesic effects of intrathecal dexmedetomidine.

Dexmedetomidine as an adjuvant to bupivacaine achieved less pain duration and intensity as postoperative VAS < 4 (min) was significantly longer in the case of bupivacaine combined with dexmedetomidine as compared to bupivacaine alone (significantly) and bupivacaine combined with meperidine (not significantly). Based on RSS between 2 and 3 (cooperative, oriented, and responsive to commands only) patients who had either bupivacaine-meperidine or bupivacaine alone had a lower level of sedation than those who had bupivacaine-dexmedetomidine. Our data is corroborated by the findings of studies conducted by S. Fyneface-Ogan. They reported that a single shot of intrathecal low dose bupivacaine/dexmedetomidine prolonged the duration of analgesia in laboring women significantly [13]. There is some clinical evidence suggesting that *α*2-adrenergic agonists enhance analgesia from bupivacaine [21].

It has been proven that *α*2-adrenergic agonists enhance analgesia from bupivacaine [18] and the efficiency of spinal bupivacaine through the action of a2-AR, which subsequently induces vasoconstriction and takes its effect in this context [22]. DEX exerts its analgesic effect through synergism with the local anesthetic or by binding to the presynaptic C-fibres and postsynaptic horn neurons [23].

In the other part, findings indicated that the combination of dexmedetomidine or meperidine with bupivacaine could decrease the incidence of shivering. Antishivering properties of the a2-adrenergic agents have been reported previously. It is proven that both intrathecal [13] and intravenous [24] DEX can decrease the incidence and intensity of shivering. The antishivering effects of dexmedetomidine are mediated by binding to *α*2-receptors in the brain and spinal cord [1] which reduces central thermosensitivity via attenuating the conductance of neurons [25].

In our study, 10 (33.3%) of patients in the BM group had an itching complication. This implies that concomitant use of meperidine with bupivacaine in this study increased the feeling of itching, whereas bupivacaine alone or in combination with dexmedetomidine had no similar effect. Chun et al. compared the group that received hyperbaric bupivacaine with the groups treated by the meperidine in terms of side effects. They found that itching complications were more prevalent in the meperidine group [26].

Hypotension was reported in all groups with no significant difference between them, indicating that there was little influence of the drug on its occurrence.

We also did not observe any cases of bradycardia. The safety of intrathecal DMT in humans has been demonstrated previously [4]. However, relatively high doses of DMT can lead to hypotension when administered intrathecal. Assistive sensory block can be achieved by intrathecal low doses of dexmedetomidine with no significant effect on blood pressure or heart rate [9, 27]. In addition, DEX, when coadministered with bupivacaine intrathecally, did not show a further decrease in blood pressure, probably because the blockade produced by bupivacaine is nearly maximum [28].

There were no signs of fetal distress in all 3 groups, evidenced by Apgar scores between 8 and 10 at 1 and 5 min, respectively, which suggests the advantageous use of dexmedetomidine over other adjuvants. Our findings were in accordance with those reported previously [11, 29] that a low dose of epidural dexmedetomidine is not known to cause significant hemodynamic effects and did not affect neonatal outcome.

## 5. Conclusion

The research reported here indicated that intrathecal administration of bupivacaine-dexmedetomidine, in comparison to bupivacaine alone and bupivacaine-meperidine, was a safe and effective analgesic option in women undergoing caesarian section. The prolonged period of sensory and motor block regression and analgesia, the minimum incidence of adverse effects such as hypotension, bradycardia, shivering and itching in the mother, and the association with good neonatal outcomes, observed in this study, could be advantages of the bupivacaine-dexmedetomidine spinal anesthesia.

## Figures and Tables

**Table 1:** Demographic and clinical characteristics.

Demographic data	Group B *n* = 30	Group BD *n* = 30	Group BM *n* = 30	*p* value
Age (years)	29.27 ± 1.08	28.93 ± 2	30.37 ± 1.1	0.610
Weight (kg)	82.63 ± 1.7	81.16 ± 1.6	79.26 ± 2.1	0.438
Systolic blood pressure base (mm·Hg)	126.56 ± 2.05	127.66 ± 1.61	123.6 ± 2.64	0.440
Heart rate base (beats/min)	97.2 ± 2.9	94 ± 2.1	98.3 ± 2.8	0.537

The values are expressed as means ± SEM. *n*, number of patients; B, bupivacaine group; BD, bupivacaine with dexmedetomidine group; BM, bupivacaine with meperidine group.

**Table 2:** Spinal block characteristics and the level of sedation.

Characteristics	Group B *n* = 30	Group BD *n* = 30	Group BM *n* = 30	*p* value
Onset of sensory block (min)	4.13 ± 1.55	4.73 ± 3.64	4.27 ± 1.78	0.698
Onset of block/motor (min) MBS = 1	5.53 ± 4.04	5.47 ± 3.83	4.80 ± 1.77	0.650
Time for sensory regression to T8 segment (min)	79.86 ± 11.12	127.73 ± 38.76^●†^	84.96 ± 22.4	<0.001
Time for motor block regression to bromage 0 (min)	111 ± 31.25	158.86 ± 34.743^●†^	120.86 ± 55.7	<0.001
Duration of VAS < 4 (min)	172.6 ± 58.9	286.53 ± 75.13^●^	259.97 ± 93.9 ^*φ*^	<0.001
Mean sedation scale	2.93 ± 0.58	2.63 ± 0.49^*∗*^	2.96 ± 0.55	<0.05

The values are expressed as means ± SEM. *n*, number of patients; B, bupivacaine group, BD, bupivacaine with dexmedetomidine group, BM, bupivacaine with meperidine group, VAS, visual analogue pain scale. ^●^*p* < 0.01 BD vs. B. ^†^*p* < 0.05 BD vs. BM. ^Φ^*p* < 0.01 BM vs. B. ^*∗*^*p* < 0.05 BD vs. B and BM.

**Table 3:** Adverse events.

Event	Group B *n* = 30	Group BD *n* = 30	Group BM *n* = 30	*p* value
Hypotension	2 (6.67)	1 (3.33)	2 (6.67)	0.809
Shivering	12 (40)^▼^	5 (16.6)	1 (3.33)	0.002
Itching	1 (3.33)	0	10 (33.3)^*∗*^	<0.001
Nausea	1 (3.33)	1 (3.33)	1 (3.33)	1
Vomiting	0	1 (3.33)	0	1

Values are expressed as number (percentage); *n*, number of patients; B, bupivacaine group, BD, bupivacaine with dexmedetomidine group, BM, bupivacaine with meperidine group. ^▼^*p* < 0.01. B vs. BM. ^*∗*^*p* < 0.01 BM vs. B and BD.

**Table 4:** Apgar scores and umbilical artery gas analysis.

Variables	Group B *n* = 30	Group BD *n* = 30	Group BM *n* = 30	*p* value
APGAR (1 min)	8.77 ± 0.63	8.73 ± 0.69	8.57 ± 0.57	0.580
APGAR (5 min)	9.53 ± 0.51	9.80 ± 4.1^∆^	9.37 ± 0.49	0.002
pH	7.36 ± 0.74	7.37 ± 0.054	7.36 ± 0.6	0.591

Values are expressed as number (percentage). *n*, number of patients; B: bupivacaine group, BD: bupivacaine with dexmedetomidine group, BM: bupivacaine with meperidine group. ^∆^*p* < 0.01 BD vs. BM.

## Data Availability

Data used in this study will be available on reasonable request to the corresponding author.
